# Joining Illumina paired-end reads for classifying phylogenetic marker sequences

**DOI:** 10.1186/s12859-020-3445-6

**Published:** 2020-03-14

**Authors:** Tsunglin Liu, Chen-Yu Chen, An Chen-Deng, Yi-Lin Chen, Jiu-Yao Wang, Yung-I Hou, Min-Ching Lin

**Affiliations:** 1 Department of Biotechnology and Bioindustry Sciences, National Cheng Kung University, Tainan, 701 Taiwan; 2 Molecular Diagnostic Laboratory, Department of Pathology, National Cheng Kung University Hospital, Tainan, Taiwan; 3 Center of Allergy and Clinical Immunology Research, College of Medicine, National Cheng Kung University, Tainan, Taiwan; 4 Department of Pediatric, College of Medicine, National Cheng Kung University, Tainan, Taiwan

**Keywords:** Metagenomics, 16S, Illumina paired-end, Taxonomy annotation, Read joining

## Abstract

**Background:**

Illumina sequencing of a marker gene is popular in metagenomic studies. However, Illumina paired-end (PE) reads sometimes cannot be merged into single reads for subsequent analysis. When mergeable PE reads are limited, one can simply use only first reads for taxonomy annotation, but that wastes information in the second reads. Presumably, including second reads should improve taxonomy annotation. However, a rigorous investigation of how best to do this and how much can be gained has not been reported.

**Results:**

We evaluated two methods of joining as opposed to merging PE reads into single reads for taxonomy annotation using simulated data with sequencing errors. Our rigorous evaluation involved several top classifiers (RDP classifier, SINTAX, and two alignment-based methods) and realistic benchmark datasets. For most classifiers, read joining ameliorated the impact of sequencing errors and improved the accuracy of taxonomy predictions. For alignment-based top-hit classifiers, rearranging the reference sequences is recommended to avoid improper alignments of joined reads. For word-counting classifiers, joined reads could be compared to the original reference for classification. We also applied read joining to our own real MiSeq PE data of nasal microbiota of asthmatic children. Before joining, trimming low quality bases was necessary for optimizing taxonomy annotation and sequence clustering. We then showed that read joining increased the amount of effective data for taxonomy annotation. Using these joined trimmed reads, we were able to identify two promising bacterial genera that might be associated with asthma exacerbation.

**Conclusions:**

When mergeable PE reads are limited, joining them into single reads for taxonomy annotation is always recommended. Reference sequences may need to be rearranged accordingly depending on the classifier. Read joining also relaxes the constraint on primer selection, and thus may unleash the full capacity of Illumina PE data for taxonomy annotation. Our work provides guidance for fully utilizing PE data of a marker gene when mergeable reads are limited.

## Background

Metagenomics has revolutionized microbiology as it bypasses the cultivation of microbes [1, 2], allowing for a comprehensive exploration of microbiota. The field has been further boosted by next-generation sequencing (NGS), which generates big data with a low cost [3]. With NGS, studying complex microbiota in various environments is now affordable for most laboratories.

Amplifying and sequencing phylogenetic marker genes, e.g., 16S ribosomal RNA (rRNA) genes, is a popular metagenomic approach with several merits. First, targeting one gene increases the sequencing depth, thus enables identification of species that constitute only a small fraction of the sample. Second, taxonomy annotation is facilitated by a wealth of reference 16S sequences of known microbes in public databases, e.g., RDP [4] and Greengenes [5].

For metagenomic studies, Illumina sequencers are popular because of their higher throughput among NGS platforms. However, Illumina reads are relatively short (150–300 bp) compared to the marker genes (e.g., ~ 1500 bp of 16S rDNAs) [6]. Fortunately, Illumina offers paired-end (PE) reads, which are sequences at the two ends of DNA fragments. When a DNA fragment is shorter than two times the read length, the paired reads overlap and can be merged into a longer read. Ideally, merged reads can reach almost two times the read length, e.g., 590 bp for MiSeq reads of 300 bp with a 10 bp minimal overlap.

Merging Illumina PE reads of a marker gene, however, can be hindered by sequencing errors. Illumina reads are prone to errors at the tail, which may inhibit identification of overlap between paired reads. For example, in many studies including ours, the variable region V3-V5 of 16S rRNA genes was amplified and the products were subjected to MiSeq 2 × 300 bp sequencing [7, 8]. For the ~ 570 bp amplicons, the majority of the PE reads could not be merged because of sequencing errors within the ~ 30 bp overlap.

**Fig. 1:**
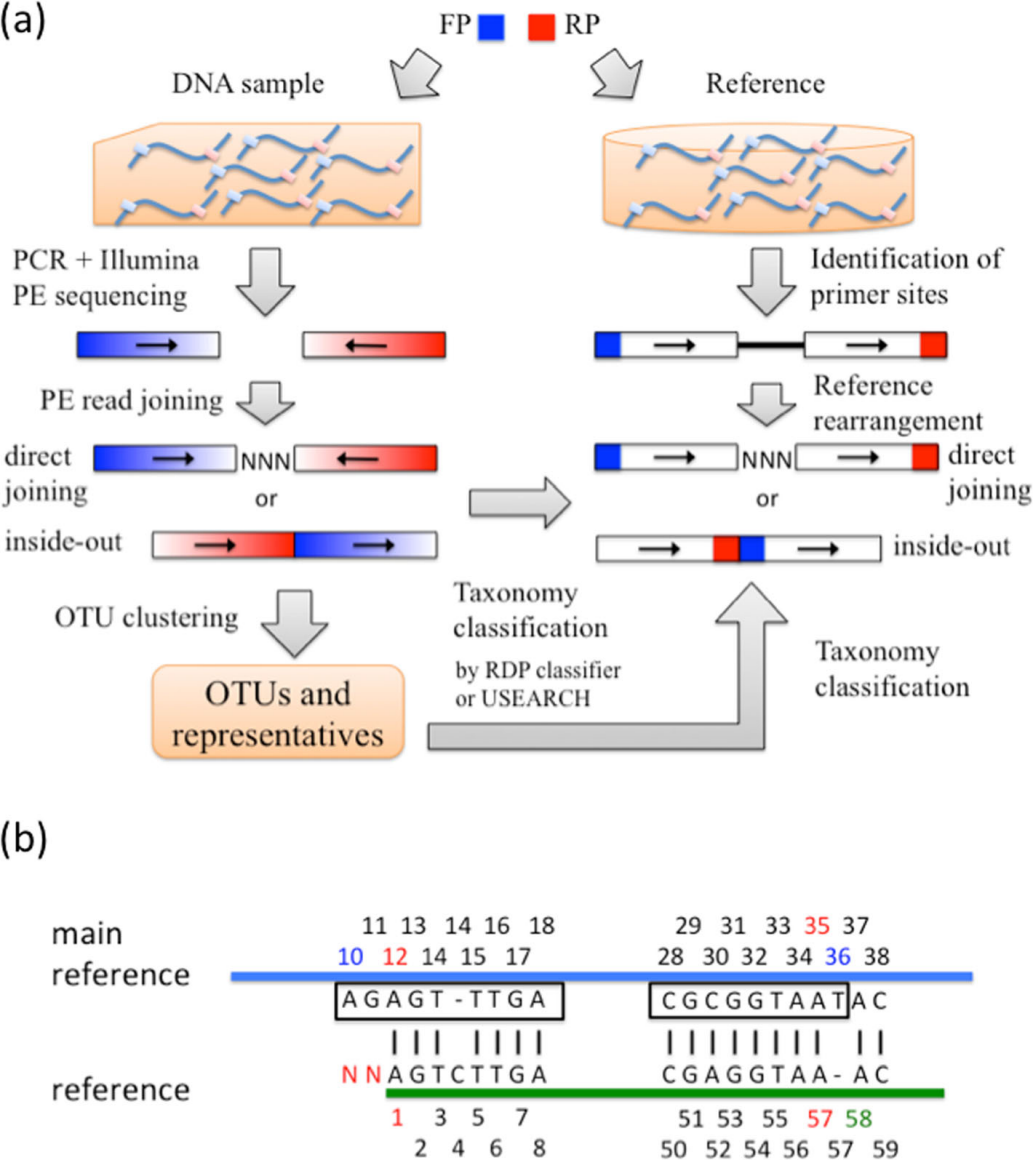
JTax workflow and reference rearrangement. **a** Given a pair of forward (blue) and reverse (red) primers, DNA samples are amplified and the products are subjected to Illumina PE sequencing (left panel). Color gradient indicates 5′ (dark; high quality) to 3′ (light; low quality) end of a read. Right and left arrows indicate reads on the plus and minus strand respectively. PE reads are then joined either directly or in an inside-out manner. The right panel shows rearrangement of a reference sequence in the two joining methods (see main text for details). **b** Identification of primer sites on a reference. Based on alignment between the main reference (blue) and a reference sequence (green), base positions on the reference are converted into coordinates on the main reference (i.e., 12, 13, 14, 14, 15, …, 34, 35, 37, 38, …). Primer sites on the main reference (in rectangles) are then obtained using USEARCH (command: search_oligo, option: –maxdiffs 3). At the forward primer site, JTax scans from the reference start the first coordinate (i.e., 12, in red) greater than the primer start (i.e., 10, in blue). To reach the primer start, it attempts to back trace two bases on the reference. However, no bases are found on the left of the corresponding position, so two Ns are padded. For the reverse primer site, the first coordinate smaller than the primer end (i.e., 36, in blue) is 35 when scanning from the reference end. JTax then extends one base from the corresponding position (i.e., 57, in red) on the reference to 58 (in green). The resulting amplicon is “NNAGTCTTGA … CGAGGTAA”

For unmergeable PE reads, one can simply just use first reads for taxonomy annotation [9]. However, this likely wastes relevant information in the second reads. To include more data, Rtax has been proposed to classify paired reads separately and then combine the annotations [10]. However, the tracking of read pairing is complicated, thus slowing down analysis [11]. In addition, the consensus strategy of Rtax has been shown to be inferior for taxonomy annotation [12]. Separate analysis of paired reads can also be done by Kraken2 [13]. However, it is mainly designed to classify whole genome shotgun data and its performance on classifying 16S data is not clear. Currently Kraken2 does not allow users to build a custom for classification. Besides separate analysis, paired reads can be concatenated (also called joined) into single reads for taxonomy annotation [14, 15]. In this work, we define “direct joining” as concatenating the reverse complement of second read RevComp(R2) to the first read (R1) with padded Ns in between (Fig. 1a). This can be done using the fastq_join command of USEARCH [16]. Another joining method is concatenating R1 to RevComp(R2), which was first proposed by Werner et al. [11]. This method is called “inside-out” by the authors as the low quality bases found in read tails corresponding to the inside of the amplicons are now placed at the two ends of the inside-out reads. Note that the inside-out method in that work was used for constructing phylogenetic tree from non-overlapping PE reads of 16S genes, not for taxonomy annotation.

Although several approaches exist for handling unmergeable PE reads, a rigorous evaluation of those approaches is still missing. Therefore, it is often not clear whether an approach is the best practice for a piece of unmergeable PE data. For example, one may suspect that sequencing errors in second reads can offset the benefit of including them because second reads usually contain more errors than first reads. In addition, different classifiers may favor different joining methods for PE reads. For example, read joining is not expected to affect much a word-counting classifier (e.g., RDP classifier [17] or SINTAX [18]). An alignment-based classifier, however, may not perform well on joined reads because of the gaps between directly joined reads and the inverted order of R1 and R2 for inside-out reads. Accordingly, reference sequences may need to be rearranged to optimize taxonomy annotation. However, to date a study of these issues has not been reported.

Here, we conducted a rigorous evaluation of the two joining methods using various simulated datasets with sequencing errors. To facilitate the evaluation, we developed a computational pipeline called JTax (**J**oining paired read for **Tax**onomy annotation). Using JTax, we assessed the benefit of joining paired reads for classification and compared the annotation accuracies by several top classifiers: RDP classifier, SINTAX, and two methods based on local and global alignment respectively. In addition, we analyzed our real Illumina PE data using various approaches to illustrate applicability of the joining methods.

Our work provides guidance for utilizing PE reads of a marker gene when the number of mergeable PE reads is limited. In our analyses of simulated and real PE data, read joining improved taxonomy annotation in general and is thus always recommended. Read joining also lifted the requirement of merging PE reads, which allows for selection of better primer pairs, e.g., those that cover more microbial species. In addition, different Illumina sequencers that generate shorter but higher quality reads may be considered. Analyzing joined reads of our real MiSeq data, we identified two bacterial genera (i.e., *Moraxella* and *Sphingomonas*) that might be associated with asthma exacerbation. The two genera are promising candidates for future exploration.

## Results

### Merge of PE reads

This work was partly motivated by the low percentages of our real PE reads that could be merged. To study relationship between airway microbes and asthma exacerbation, we collected nasal samples from 12 asthmatic children and explored the microbial communities (Methods). Briefly, 16S segments were amplified using the Human Microbiome Project primer pairs 27F/534R and 357F/926R (Table 1), which probes the V1-V3 and V3-V5 regions respectively for MiSeq 2 × 300 bp sequencing. For the V1-V3 primer pair, only 1,739,397 of the 3,559,206 PEs (48.9%) could be merged by USEARCH with a 25% maximal mismatch rate within overlap (Table S1). A majority of the unsuccessful merges could be attributed to sequencing errors because most paired reads were expected to overlap by ~ 90 bp. For the V3-V5 primer pair, only 48,925 of the 320,169 PEs (15.3%) could be merged. This is reasonable as the longer amplicons resulted in shorter overlaps and higher mismatch rates within the overlaps.

**Table 1:** Primer pairs used in this study and the covered variable regions

Primer pair	Forward/ reverse sequence	Covered variable region
27F/534R	AGAGTTTGATCCTGGCTCAG/ ATTACCGCGGCTGCTGG	V1-V3
357F/926R	CCTACGGGAGGCAGCAG/ CCGTCAATTCMTTTRAGT	V3-V5
341F/785R	CCTACGGGNGGCWGCAG/ GACTACHVGGGTATCTAATCC	V3-V4
341F/1062R	CCTACGGGNGGCWGCAG/ CRRCACGAGCTGACGAC	V3-V6
8F/785R	AGRGTTYGATYMTGGCTCAG/ TACHVGGGTATCTAAKCC	V1-V4
27F/1492R	AGRGTTYGATYMTGGCTCAG/ RGYTACCTTGTTACGACTT	V1-V9

Note that he primer coordinates are adapted from the original references, which might apply different coordinate systems

The problem of unmergeable PE reads has occurred in many projects [7, 8]. For example, on 2019 Nov 1, the NCBI Sequence Read Archive (SRA) hold MiSeq PE data of at least 2672 samples in 33 metagenomics projects that probed the V3-V5 regions. For more than half of the samples, we estimated that less than half of the PE reads could be merged (Table S2). For those projects, paired reads can be joined into single reads, e.g., using JTax, for taxonomy annotation.

### JTax workflow

The main task of JTax is rearranging reference sequences in a direct joining or inside-out manner (Fig. 1a) for classifying the corresponding joined reads. Given a primer pair and a reference database containing full-length sequences of 16S or another marker gene, JTax first extracts amplicons via identifying primer sites on the references (see below). At the two ends of the amplicons, segments of the corresponding read lengths are extracted respectively and joined directly or in the inside-out manner as the rearranged references for several classifiers. JTax incorporates two word-counting classifiers, the RDP classifier (v2.12) (RDP) and SINTAX (in USEARCH v11.0.667), and two top-hit methods based on global alignment by USEARCH (v11.0.667) (TOP) and local alignment by BLAST (v2.9.0+) (BTOP) respectively. JTax is designed to be modular and includes a module to join PE reads. Before joining, JTax can trim primer from reads and correct sequencing errors within overlap of paired reads if some overlap is still expected. The modular fashion of JTax facilitates comparison of joined reads to a different reference database, e.g., amplicon sequences.

To identify primer sites on reference sequences, JTax first selects a main reference. Specifically, reference sequences that contain a unique binding site of the forward and reverse primers are identified. The reference with the longest segments outside the binding sites is selected as the main reference. JTax then aligns all reference sequences to the main reference, and converts base positions on those sequences into coordinates on the main reference (Fig. 1b) for identifying the corresponding primer sites. If a reference does not extend to the primer site, JTax pads Ns until reaching 5′ end of the primer. This saves reference sequences falling short at the primer sites. Implementation details can be found in the JTax codes.

### Benefit of including second reads for taxonomy annotation

We set out to examine whether joining unmergeable PE reads could improve taxonomy annotation. Our evaluation was inspired by the TAXXI paper [12], in which the idea of cross-validation by identity was first introduced. Briefly, it was observed that a majority of real metagenomic 16S sequences did not have a highly similar counterpart (e.g., with an identity > 99%) in the authentic reference database. For more realistic benchmarking, training and testing data should be prepared such that the top-hit identities of the test sequences cover different values to mimic the trend of real data. Please refer to the TAXXI paper for more details. Along this line or reasoning, we designed a greedy algorithm to split a reference database into a pair of training and testing sets of sequences with a desired top-hit identity (e.g., 97%) for the V3-V5 primer pair (Methods). The algorithm optimized the number of testing and training sequences under the constraint of top-hit identity for better statistics. To build training and testing datasets, we used the NCBI BLAST 16S rRNA (BLAST16S) sequences from the TAXXI paper because that data is from authoritatively isolated strains. From the testing sequences, MiSeq 2 × 300 bp reads were simulated based on the quality profiles of our real MiSeq data (Fig. S1).

The simulated first reads, directly joined reads, and inside-out reads were then compared to the training sequences for annotation by two word-counting classifiers (RDP and SINTAX) and two alignment-based top-hit methods (TOP and BTOP). Those classifiers were selected for their top performance in the TAXXI paper. Note that the training sequences were amplicons of the V3-V5 primer pair. In addition to the amplicons, the two types of joined reads were compared to the correspondingly rearranged references respectively for taxonomy prediction. Classification accuracy at each top-hit identity (100, 99, 97, 95, and 90%) and the mean value were then calculated (Methods). The two word-counting classifiers provide confidence score and two thresholds, 50 and 80%, were used for accuracy estimation.

**Fig. 2:**
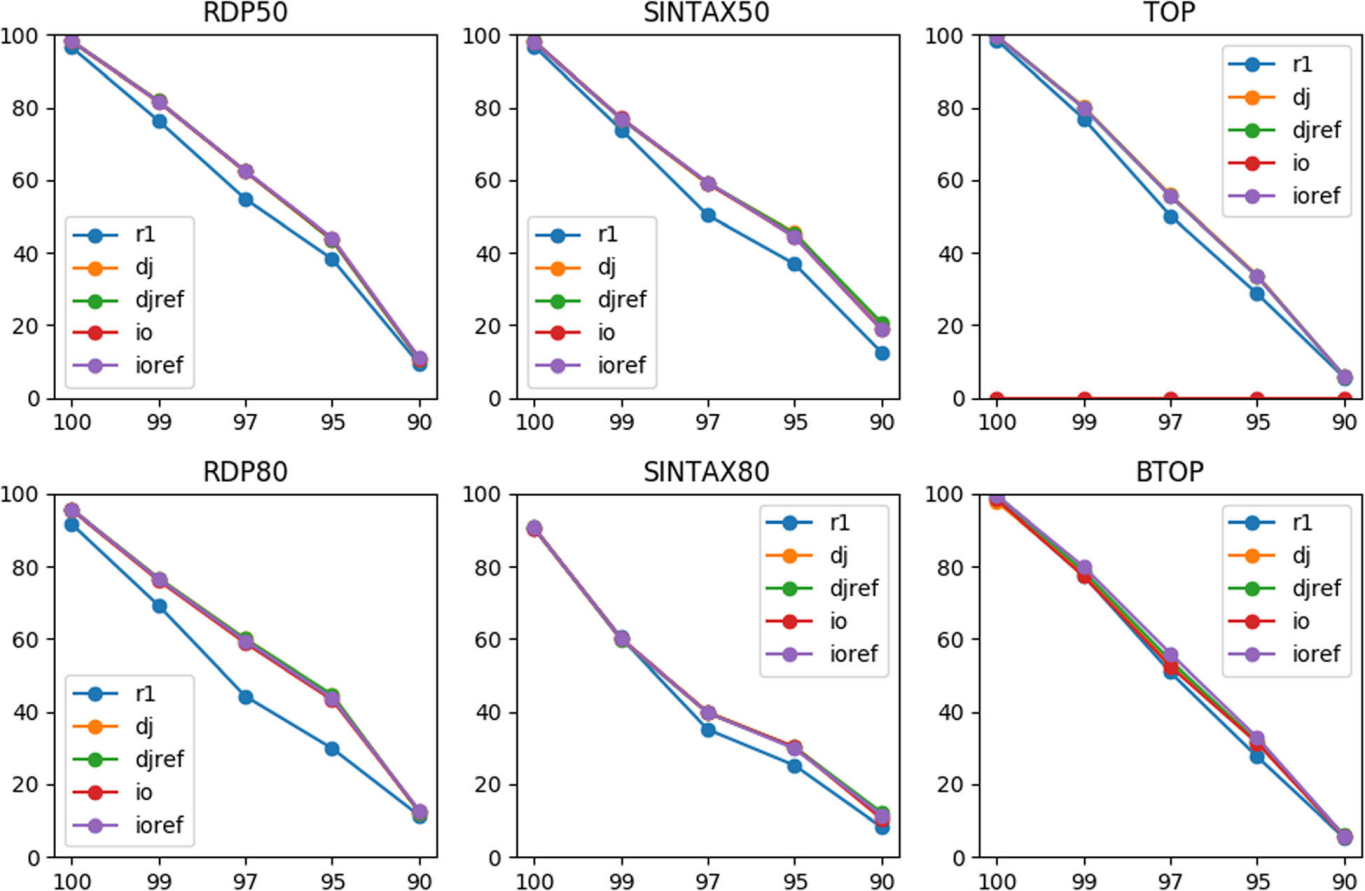
Accuracies (y-axis) of classifying different types of reads for the V3-V5 primer pair at various top-hit identities (x-axis). Three types of reads, first (r1), directly joined (dj), and inside-out (io) reads, were classified to the genus level at different top-hit identities by six classification methods using MiSeq 2 × 300 bp data. The directly joined and inside-out reads were also compared to the corresponding rearranged references (djref and ioref) respectively for classification

For all classifiers, including second reads resulted in a similar or higher genus level accuracy at all top-hit identities except for the TOP classification of inside-out reads using amplicons as reference (Fig. 2). The overall improvement indicates that sequencing errors in the simulated second reads did not offset the benefit of including them for taxonomy annotation.

TOP failed to classify all inside-out reads because the reads could not be aligned well globally to the amplicons due to the inverse order of R1 and R2. This serious fault could be fully rescued via rearranging the training sequences in the inside-out manner. Such a problem was less serious for local alignments by BTOP because still half of the inside-out reads, i.e., either the first or second reads, would be aligned to the amplicons with the other half omitted. The resulting performance was thus at least as good as using first reads, but the advantage of PE data could not be exploited (Fig. 2). Again, local alignments of inside-out reads to the inside-out references improved the mean accuracy from 53.1 to 54.9% (Table 2) as the whole reads could be aligned. Thus, reference rearrangement clearly can affect the performance of alignment-based classifiers. In contrast, the word-counting classifiers were not affected by reference rearrangement for this primer pair. For RDP and SINTAX, joined reads achieved higher true positive rates and lower under-classification rates compared to first reads in general (Fig. S2). But over-classification rates of joined reads were higher using RDP for classification. For the two top-hit classifiers, read joining also increased true positive rates while lowering misclassification rates.

**Table 2:** Mean accuracies of classifying different types of reads for the V3-V5 primer pair

Read	RDP50	RDP80	SINTAX50	SINTAX80	TOP	BTOP
r1	55.1	49.34	54.12	43.9	52.02	52.02
dj	59.34	57.9	59.82	46.4	55.08	53.46
djref	59.32	57.92	60.08	46.54	54.94	54.04
io	59.38	57.34	59.5	46.24	0	53.08
ioref	59.52	57.7	59.56	46.36	54.94	54.86

Three types of reads, first (r1), directly joined (dj), and inside-out (io) reads, were classified to the genus level by six classification methods using MiSeq 2 × 300 bp data. The directly joined and inside-out reads were also compared to the corresponding rearranged references (djref and ioref) respectively for classification

**Fig. 3:**
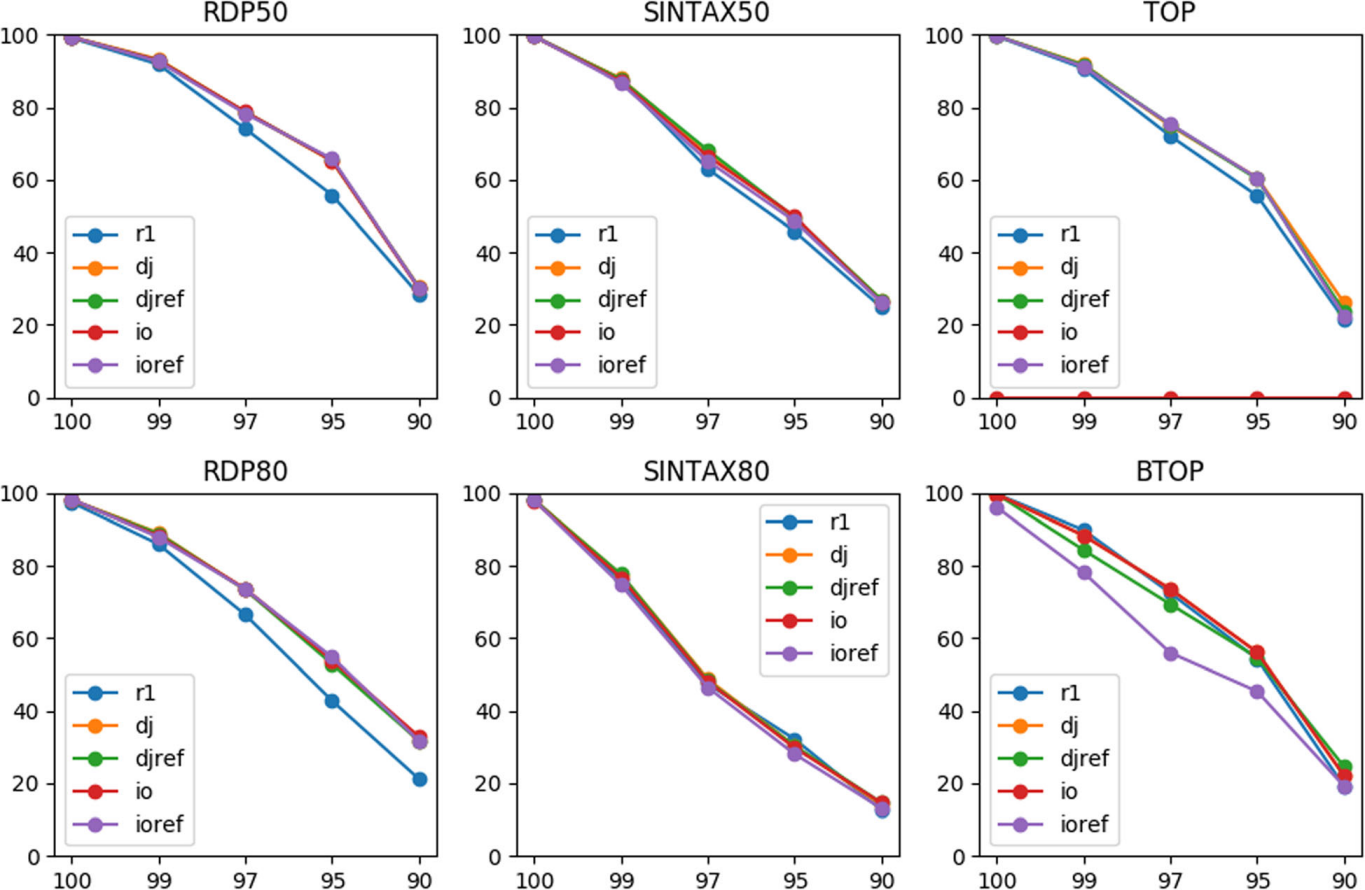
Accuracies (y-axis) of classifying different types of reads for the V1-V3 primer pair at various top-hit identities (x-axis)

We repeated the above analyses for the V1-V3 primer pair (Table 1). Similar results were observed except for the smaller improvement using SINTAX and the declined performance using BTOP when inside-out reads were compared to the inside-out references (Fig. 3, Table 3, and Fig. S3). We found that many training sequences did not extend to the primer site 27F, which resulted in padded Ns for the missing segments in the middle of inside-out references, which might break the local alignments. As a consequence, the references with no or fewer padded Ns were favored for alignment, so this bias may explain the lower accuracy. Padded Ns were less of an issue for TOP because they could not break global alignments. This explains the better performance of TOP than BTOP in general. For the word-counting classifiers, reference rearrangement again did not make a difference in accuracy. Considering these results, we recommended always joining unmergeable PE reads for taxonomy annotation.

**Table 3:** Mean accuracies of classifying different types of reads for the V1-V3 primer pair

Read	RDP50	RDP80	SINTAX50	SINTAX80	TOP	BTOP
r1	69.82	62.8	64.18	53.36	67.9	67.14
dj	73.38	69.32	66.24	53.78	70.62	67.98
djref	73.32	69	66.38	53.8	70.1	66.58
io	73.26	69.4	65.86	53.44	0	67.98
ioref	73.26	69.26	65.26	52.12	69.86	59

### Toward full capacity of PE reads for taxonomy annotation

The effectiveness of read joining implies that the requirement of merging PE reads can be lifted. This motivated us to study whether increasing non-redundant informative bases via probing longer 16S segments could improve taxonomy annotation. To this end, we simulated PE reads for two other primer pairs that covered the V3-V4 and V3-V6 regions respectively (Table 1). The V3-V4 primer pair was recommended by Klindworth et al. [19] and the Illumina company. The V3-V6 primer pair was also recommended by Klindworth et al. for its higher bacterial coverage but slightly lower phylum coverage compared to the V3-V4 primer pair. For the V3-V4 primer pair, most (> 99.9%) of the simulated PE reads could be merged as the ~ 450 bp amplicons led to an ~ 150 bp overlap for the 300 bp paired reads, and sequencing errors within the overlaps could be corrected. In contrast, none of the paired reads of the V3-V6 amplicons (~ 725 bp) overlapped. Therefore, all 600 bases in the PE reads provided non-redundant information for taxonomy prediction, but sequencing errors in the reads could not be corrected. Here, we compared merged reads of the V3-V4 amplicons with the directly joined and inside-out reads of the V3-V6 amplicons for taxonomy prediction.

**Fig. 4:**
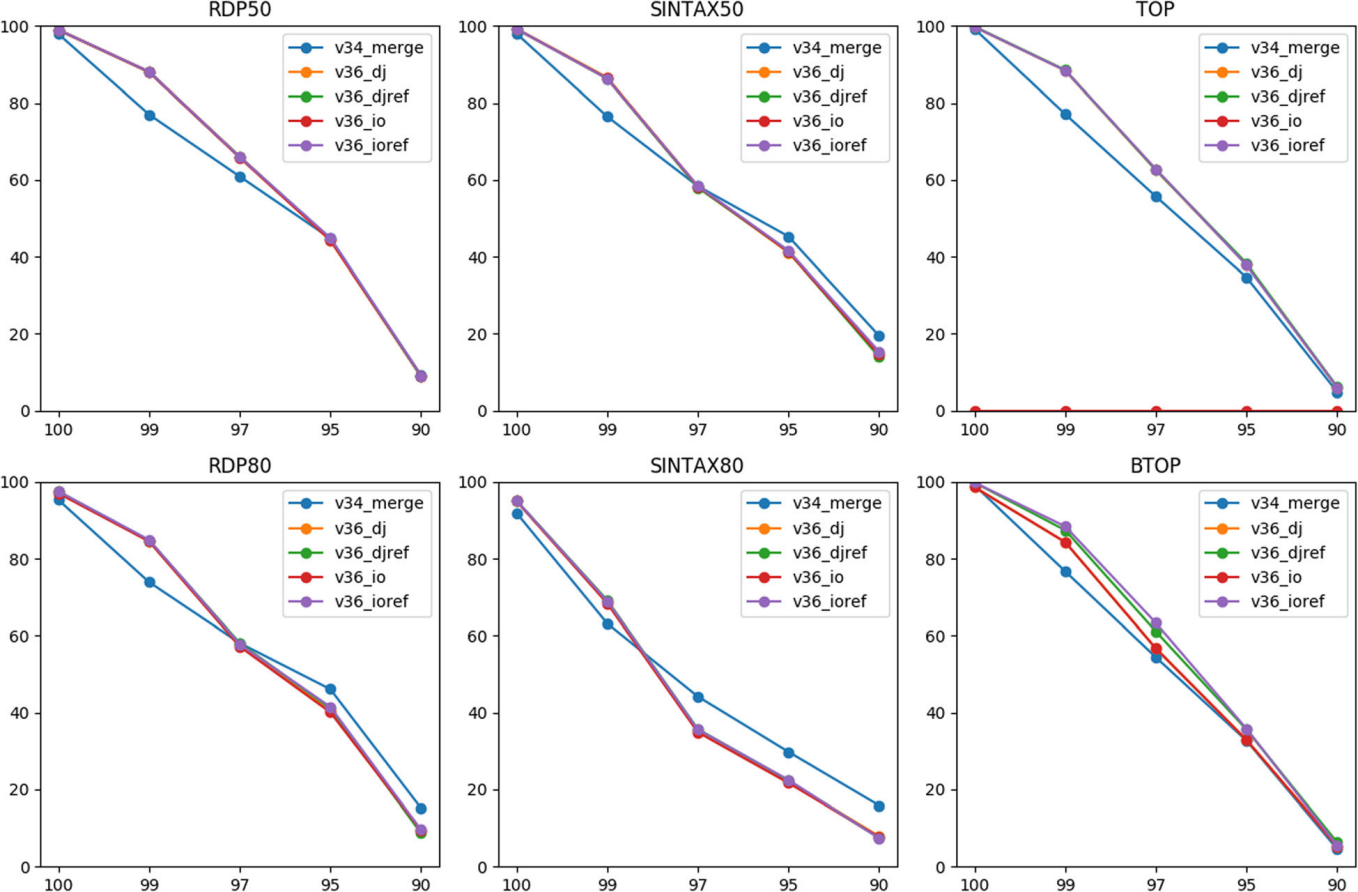
Classification accuracy (y-axis) for the V3-V4 merged reads and V3-V6 joined reads (MiSeq 2 × 300 bp) at various top-hit identities (x-axis)

Figure 4 reveals that the V3-V6 joined reads achieved a similar or better genus level accuracy compared to the V3-V4 merged reads using RDP50, TOP, and BTOP for classification at all top-hit identities. For RDP80 and SINTAX, the accuracies of the V3-V6 joined reads were higher than the V3-V4 merged reads at the top-hit identities 100 and 99%, but lower when the testing data were less similar to the training data. In terms of mean accuracy, the V3-V6 joined reads were comparable to or better than the V3-V4 merged reads for all classifiers except SINTAX80 (Table 4). The lowest performance of SINTAX80 was consistent with the TAXXI paper. Note that RDP50 achieved the highest mean accuracy on the V3-V6 joined reads among all classifiers and data types. This indicates that additional informative bases in the V3-V6 joined reads could compensate the downside of sequencing errors and even improve taxonomy prediction for some classifiers.

**Table 4:** Mean classification accuracy for the V3-V4 merged reads and V3-V6 joined reads (MiSeq 2 × 300 bp)

Read	RDP50	RDP80	SINTAX50	SINTAX80	TOP	BTOP
v34_merge	57.96	57.72	59.54	49	54.32	53.5
v36_dj	61.3	57.92	60.06	45.8	58.94	55.56
v36_djref	61.3	58.02	59.78	45.96	59.08	58.04
v36_io	61.22	57.64	59.96	45.54	0	55.56
v36_ioref	61.42	58.26	60.18	45.92	58.96	58.56

For the V3-V6 joined reads, the two joining methods achieved a similar mean accuracy using RDP and SINTAX for classification and reference rearrangement again did not make a difference. For TOP and BTOP, the two types of joined reads also achieved a similar accuracy when they were compared to the corresponding rearranged references. As expected, using amplicons as reference could lower performance of the alignment-based classifiers. For example, BTOP accuracies of the joined reads were lower when being compared to the amplicons because only half of the reads were locally aligned.

### Possibility of applying a different Illumina platform

The benefit of increasing informative bases is expected more obvious if sequencing error rate is lower. In other words, with fewer sequencing errors, a smaller increase of informative bases may achieve a similar degree of improvement on classification. To examine this hypothesis, we obtained a real HiSeq 2 × 250 bp dataset, which showed a higher quality than our MiSeq data (Fig. S1), and repeated the above analyses.

**Fig. 5:**
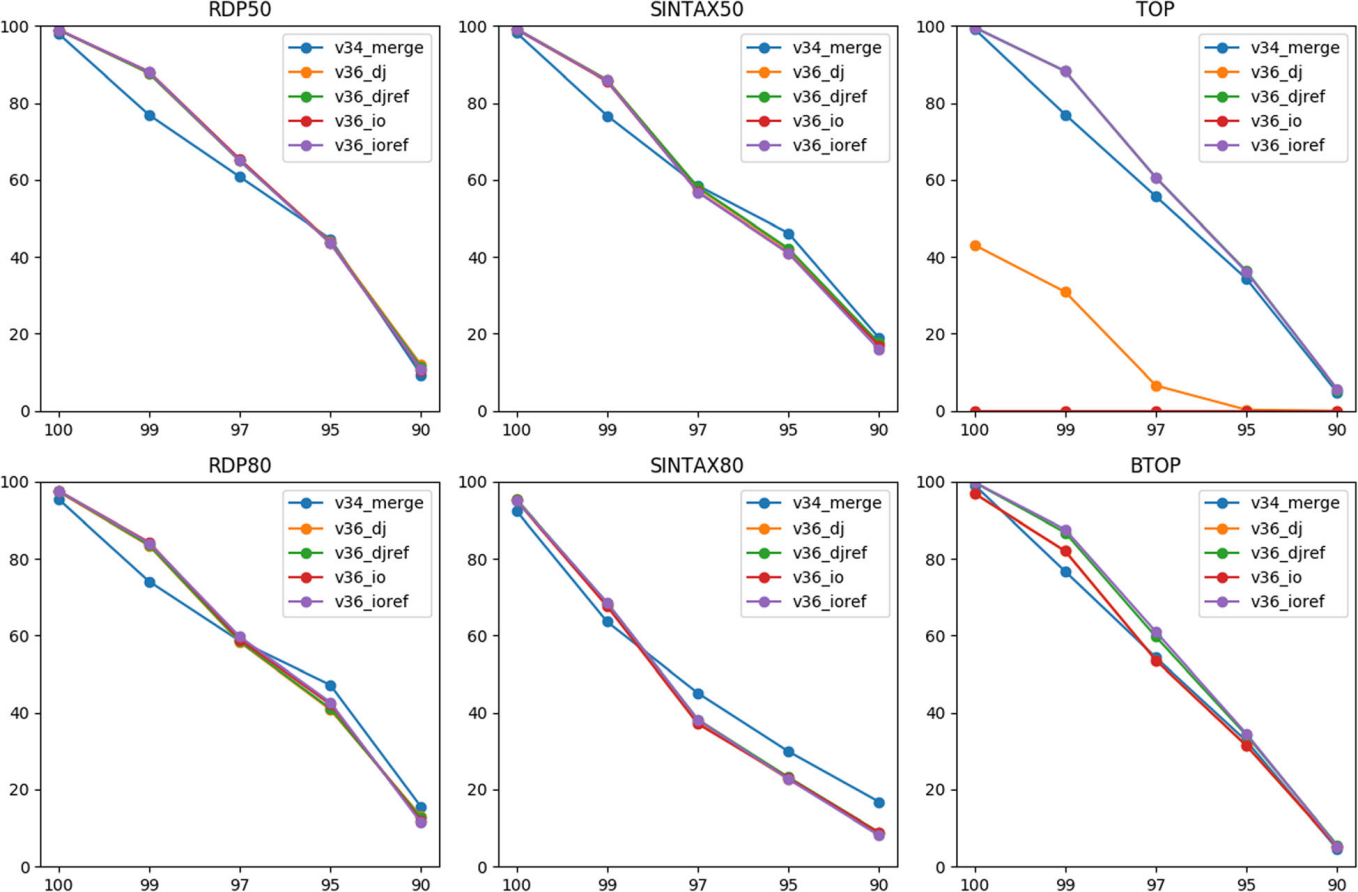
Classification accuracy (y-axis) for the V3-V4 merged reads and V3-V6 joined reads (HiSeq 2 × 250 bp) at various top-hit identities (x-axis)

For the V3-V4 region, most simulated PE reads of 250 bp were still long enough to be merged. The mean accuracies of merged reads, however, remained similar compared to the simulated MiSeq data (Table 5 and Fig. 5). This suggests that error correction via overlap between the MiSeq paired reads was already effective. For the V3-V6 region, the joined HiSeq and MiSeq reads achieved a similar mean accuracy for the two word-counting classifiers. This confirms our presumption that 500 bp joined reads with a higher quality is enough to improve the classification accuracy to the same degree as done by the 600 bp joined reads with a lower quality.

**Table 5:** Mean classification accuracy for the V3-V4 merged reads and V3-V6 joined reads (HiSeq 2 × 250 bp)

Read	RDP50	RDP80	SINTAX50	SINTAX80	TOP	BTOP
v34_merge	57.96	58.18	59.7	49.56	54.2	53.46
v36_dj	61.52	58.58	60.46	46.52	16.18	53.8
v36_djref	61.36	58.7	60.6	46.82	58.04	57.2
v36_io	61.36	59	59.9	46.32	0	53.8
v36_ioref	61.28	59.1	59.74	46.56	58.04	57.64

Notably, the mean accuracy of classifying directly joined V3-V6 reads by TOP dropped from 58.9% (MiSeq) to 16.2% (HiSeq) when using amplicons as reference. This drop could be explained by larger gaps (~ 225 bp) between the paired HiSeq reads of 250 bp. The large gaps decreased identities of the global alignments and disturbed the ranking significantly. This demonstrates that classifying directly joined reads via global alignment to the amplicons could be affected by read length. Again, rearranging the reference sequences in the direct joining manner restored the accuracy.

### Analysis of our real MiSeq PE data

To demonstrate applicability of read joining, we analyzed our MiSeq PE reads of nasal microbiota in the 12 asthmatic children. For each child, nasal microbes were sampled during asthma attack and in the recovery phase (Methods). Via comparing microbiota in the two phases, we searched for microbes correlated with asthma exacerbation. As mentioned above, more than half (55.2%) of the V1-V3 PE reads could not be merged with a 25% maximal mismatch rate (Table S1). We therefore joined the PE reads in both the direct and inside-out methods for analysis. The V3-V5 data were not analyzed because the amount was much smaller.

Before joining, both first and second reads were trimmed to reduce the impact of sequencing errors. To optimize classification, 20 bp were trimmed from tail for all first reads gradually until the trimmed reads achieved a maximal mean confidence score at the genus level by RDP classifier using the RDP full-length 16S training sequences as reference. Similar optimization was done for the second reads. The optimal lengths of trimmed first and second reads were 260 bp and 160 bp respectively, and most paired trimmed reads could not be merged.

We analyzed four types of data: the 3,559,206 trimmed first reads, the two types of joined trimmed reads, and the original 1,738,393 merged reads longer than 290 bp. For each data type, reads were first clustered into operational taxonomic units (OTUs) (Methods) and the OTU representative sequences were annotated by RDP classifier. This procedure was efficient and helped correct sequencing errors; the obtained OTUs also facilitated community analysis. Note that the OTU analysis failed to cluster joined reads without trimming (see Discussion).

The three types of trimmed reads resulted in more OTUs than the merged reads (Table 6). In terms of data usage, more joined reads were used for inferring OTU abundance compared to the merged data. For example, 2,479,548 of the directly joined reads could be mapped to the corresponding OTUs while only 1,526,567 merged reads could be mapped. We considered the mapped reads belonging to confident OTUs (genus level confidence score ≥ 0.8) as effective, and found that more joined reads were effective than other two types of data. These indicate that read joining made better use of the real PE data for taxonomy annotation. In the following analyses, directly joined reads were used.

**Table 6:** Statistics of clustering different types of our real MiSeq data into OTUs

	No. of reads	No. of OTUs	No. of reads mapped to OTUs	No. of confident OTUs	No. of reads in confident OTUs
Merged	1,738,393	153	1,526,567	103	1,431,993
Trimmed first	3,559,206	395	2,542,518	268	2,327,060
Trimmed DJ	3,559,206	344	2,479,548	250	2,459,159
Trimmed IO	3,559,206	309	2,430,418	232	2,411,575

*Abbreviation*s: *DJ* Direct-joining, *IO* Inside-out

To investigate whether asthma status affected microbiota, we compared the asthma attack and recovery samples using UniFrac [20] (Methods). Principal coordinate analysis revealed that asthma status was not a major factor for shaping the community structure (Fig. S4). In addition, the weighted UniFrac distances between two samples of the same individuals were significantly smaller than distances between samples of different individuals (T test *p*-value 0.027). This suggests individual difference and that the asthma attack and recovery samples of the same individuals should be compared. Specifically, we looked for OTUs that showed a higher or lower proportion (by ≥3%) during asthma attack and the number of cases in one direction was greater by at least three fold and three compared to the other direction. This criterion identified two OTUs (Otu2 and Otu7) and the corresponding genera were *Moraxella* and *Sphingomonas* respectively (Table S3). The two OTUs showed a higher proportion in the noses of four and three patients and lower in none respectively. Interestingly, those two genera have been associated with child asthma (see Discussion), and therefore are promising for further experimental investigation.

Repeating the above analysis for the trimmed first reads failed to identify any differential OTU. For the merged reads, only *Moraxella* but not *Sphingomonas* was identified. This demonstrates the benefit of joining PE reads when their merges are limited, as in 16S studies.

## Discussion

### Correction of sequencing errors

Our simulation demonstrated that joining unmergeable PE reads could improve taxonomy annotation. The estimated benefit is conservative because we did not consider the possibility of correcting sequencing errors. In 16S studies, sequencing errors can be corrected via referring to other sequences in the data [21, 22]. For example, clustering sequences into OTUs is also an act of error correction and the OTU representative sequences are usually highly accurate, which should enhance the benefit of read joining. Indeed, we repeated the simulated comparison of the V3-V4 merged reads and V3-V6 joined reads using error-free MiSeq 2 × 300 bp reads, and found a greater improvement in taxonomy prediction (Fig. S5 and Table S4).

Although error correction is possible, we emphasize the importance of trimming low quality bases when analyzing real data. In our data, for example, if the whole first and second reads were joined directly, almost all joined reads would fail to pass the filtering step of OTU clustering, which keeps only reads with less than one expected error. We tried increasing the threshold to ten, but almost all reads passing the filter are singletons, which seriously deteriorated the OTU clustering (only ten OTUs were obtained). Therefore, trimming low quality bases is necessary to ensure an appropriate OTU clustering. Note that we suggest trimming reads to a fixed length instead of quality trimming for OTU clustering. Quality trimming usually results in trimmed reads of different lengths, which biases the clustering procedure [21].

### Full-length sequences and amplicons as reference

Confining reference sequences to the amplicon region has been shown to improve taxonomy annotation [23]. For our real data, limiting references to the amplicon region indeed gave more confident OTUs at the genus level, e.g., from 250 to 255 with the directly joined reads. Although the improvement is not large, using amplicons as reference is usually favored. To extract amplicons, identifying primer sites via aligning primer to reference will fail if the reference sequences do not extend to the primer site. For example, among the 13,212 training sequences in the RDP 16S database, only 3113 covered the 27F primer site. JTax addresses this issue via selecting a long sequence that covers both primer sites as the main reference and extracting amplicon based on pairwise alignment between each reference sequence and the main reference. For the V1-V3 primer pair, JTax output 13,206 amplicons and missed only six sequences because the bases did not make up at least half of the amplicons.

### Potential microbes associated with asthma exacerbation

We identified *Moraxella* and *Sphingomonas* as candidate bacterial genera associated with asthma exacerbation in children. Consistently, those bacteria have been implicated in childhood asthma. For example, in acute respiratory illness, which is mainly caused by viral infection, *Moraxella* was also found to be more abundant in the nasopharynx of patients [24]. In fact, the causal effect of *Moraxella* in asthma exacerbation has recently been shown via animal experiments [25]. This suggests that the *Moraxella* species in the noses of some patients likely triggered the asthma exacerbation. The genus *Sphingomonas* has been reported to be enriched in the house dust of children with asthma [26]. This may explain the enrichment of *Sphingomonas* in the noses of some asthmatic children during asthmatic attack. In bronchial microbiome studies of asthmatic patients, the family Sphingomonodaceae has also been shown to be enriched [27] and highly correlated with the degree of bronchial hyper-responsiveness [28]. These corroboraing reports support validity of our experimental and analytical procedures.

## Conclusions

In metagenomic studies involving a marker gene, Illumina PE reads sometimes cannot be merged for taxonomy annotation. Face with this problem, it is often not clear how to use the PE data effectively because a detailed evaluation of different approaches has been missing. Here, we rigorously evaluated procedures to utilize unmergeable PE data for classification by various top classifiers. Based on our results we make several suggestions. First, joining PE reads into single reads is always recommended as read joining improved the classification accuracy in most of our investigations with simulated sequencing errors. Second, trimming reads to a fixed length before joining is suggested to optimize OTU clustering and classification. Third, the joining method (direct joining or inside-out) can affect performance of alignment-based classifiers, but not word-counting classifiers. For alignment-based classifiers, rearranging reference is recommended to avoid problems caused by gaps between or the inverse order of paired reads. In general, a classifier based on global alignment is favored over one based on local alignment because the whole joined reads (i.e., all available information) are used in global alignment. For word-counting classifiers, rearranging the reference sequences did not make a difference in classification accuracy. Therefore, joined reads can be directly compared to the original reference database. To further improve classification, amplicons instead of full-length sequences can be used as reference, although the improvement may be minor. Amplicon extraction will fail when reference sequences do not extend to the primer site, but this can be rescued by JTax. To join PE reads, direct joining using fastq_join in USEARCH is recommended if no primer removal or error correction is desired. Otherwise, JTax can be used. These recommendations should be useful for properly utilizing unmergeable PE data of a marker gene in metagenomic studies. JTax is written in Perl and is freely available in Github (https://github.com/TLlab/JTax).

## Methods

### Data for evaluating taxonomy prediction

Full-length 16S sequences with known taxonomy (i.e., the file ten_16s.100) were obtained from the TAXXI [12] benchmark data. The reference sequences were a subset of the NCBI BLAST 16S rRNA database (July 1, 2017), in which at most ten sequences per genus were randomly selected and kept. This alleviated the concern of unbalanced reference for performance evaluation.

To implement the idea of cross-validation by identity, we designed a greedy algorithm to partition the TAXXI reference into training and testing datasets such that the alignment identity between each testing sequence and the best hit in the training data was within a certain range, e.g., 97 ± 0.5%. Readers are suggested to consult Fig. 1 of the TAXXI paper first to understand cross-validation by identity before going through the following procedure, which employs similar notations.

Given reference sequences and a primer pair, the corresponding amplicons were first extracted using JTax as confined references. All pairs of amplicons were then aligned globally using USEARCH [16] (v11.0.667) and the alignment identities were obtained.

With the confined references (R), our greedy algorithm attempted to optimize the number of references in the testing (S) set, which also determined the top hits (T) that were within the specified range of identity d ± δ%. Following the TAXXI paper, we used δ = 0.5 for d = 99,97,95 and δ = 1.0 for d = 90. For d = 100, a natural choice was to use R as both the training and testing datasets. For d < 100, we first defined the hits of a reference r with an identity greater and within the specified range as z(r) and t(r) respectively. If a reference r was assigned to S, then z(r) should be excluded from the training set (A) and assigned to the excluding set (Z) while those with an identity <d-δ (defined as in the set W) could stay in A, which is therefore union of T and W. As optimizing S and A was similar to minimizing Z, a reference r should be assigned to S or A earlier if it excluded fewer sequences. Moreover, existing references in A limit the chance for an r to be added to S because some references in A might have an identity to r greater than d + δ. Therefore, it was better to increase A slowly. Based on these ideas, for each reference r we defined tz(r) as the union of z(t) where t represented the top hits of r within the identity range. We then sorted the references by z(r) and tz(r) from small to large.

Starting with empty S, A, and Z, the first reference r was assigned to S, and the t(r) and z(r) were assigned to A and Z respectively. For the next reference r, if at least one of the t(r) had not been assigned to S or Z, r was assigned to S and the non-assigned t(r) were assigned to A. Otherwise, r was assigned to A if it had not been assigned to Z. To increase A slowly, the number of references assigned to A was limited to no more than three in each run. This procedure was repeated for all references. At the end, all reference r’s that had not been assigned to any set (i.e., no hit above or within the identity range) were assigned to A. The resulting A and S served as the training and testing datasets for evaluating taxonomy prediction. Note that for the V1-V3 primer pair, some references did not extend to the primer site 27F, thus could only serve as training data but not testing data.

For each testing dataset, we simulated MiSeq 2 × 300 bp or HiSeq 2 × 250 bp reads using ART (MountRainier-2016-06-05) [29]. The simulation used quality profiles built from our real MiSeq dataset and one HiSeq dataset from NCBI SRA (SRP136977). For each testing sequence, three PEs were simulated.

### Performance metrics of taxonomy prediction

We followed the TAXXI paper to evaluate performance of taxonomy prediction. For the hierarchical nature of taxonomy annotation, we calculated three types of errors: over-classification (OC), under-classification (UC), and misclassification (MC) rates at different taxonomy levels. At a level, an OC error occurred when the predicted rank did not exist in the training data. An UC error occurred when the test sequence’s rank that also existed in the training data was not predicted. Let TP be the number of test sequences whose rank was correctly predicted, K be the number of test sequences whose rank existed in the training data, and OC also be the number of OC errors, accuracy of prediction was defined as TP/(K + OC). For cross-validation by identity, mean accuracy of the five top-hit identities was also calculated. Please refer to the TAXXI paper for definitions of other performance metrics. The metrics were calculated using scripts from the TAXXI paper.

### Patient recruitment and study design for the role of airway microbes and asthma exacerbation

Asthmatic children aged 5 to 12 years with recurrent wheeze were recruited. Exacerbated asthma without fever was defined as self-reported and physician-diagnosed current asthma presenting with a chief complaint of shortness of breath with an encounter diagnosis and need acute reliever treatment of asthma exacerbation. Non-exacerbated asthma was defined as self-reported and physician-diagnosed current asthma presenting for routine, non-urgent, asthma follow-up care.

### Sample collection and processing

We collected samples in duplicate using sterile cotton swabs from anterior nares of nasal cavities and retropharyngeal space of 12 asthmatic children at both acute asthma exacerbation and recovery phase (2-week apart). Swabbed samples were kept in 1.5 ml sterile saline buffer for microbiome analysis.

### DNA extraction

All of the swab samples were transported to the core facility with ice packs within 1 h after collection. Samples were vortexed for 30 s at room temperature in 1 ml sterile saline solution. After centrifugation at 12000 rpm for 10 min, the supernatant was discarded and the pellet re-suspended in 50ul *RNAlater*. From the nasal cavity and throat suspensions, DNAs were extracted by QIAamp DNA Microbiome Kit (Qiagen). The DNA extraction was performed according to the manufacturer’s instructions. All extracted DNA samples were stored at − 80 °C until further processing.

### Amplification of the 16S region

The polymerase chain reaction (PCR) amplifications were performed under the following conditions: initial denaturation at 95 °C for 3 min, followed by 40 cycles of denaturation at 95 °C for 30 s, annealing at 55 °C for seconds, and extension at 72 °C for seconds, final extension at 72 °C for 5 min. Quantity and quality of the extracted DNA were analyzed by spectrophotometry using NanoDrop 2000 Spectrophotometer (Thermo Scientific) and by agarose gel electrophoresis. PCR clean-up used AMPure XP beads to purify the 16S amplicons to remove free primers and primer dimmers.

### Library preparation and sequencing

Sequencing libraries were generated using TruSeq® DNA PCR-Free Sample Preparation Kit (Illumina, USA) following manufacturer’s recommendations. Index codes were added to Illumina sequencing adapters and dual-index barcodes to the amplicon target. The library quality was assessed on Qubit 2.0 Fluorometer (Thermo Scientific) and Agilent 2100 Bioanalyzer system. The products were then subjected to 2 × 300 bp PE sequencing on MiSeq.

### OTU analyses

Clustering of 16S reads was done by UPARSE [21] in USEARCH (v11.0.667) as follows. First, low quality reads of all samples were filtered (command: fastq_filter, option: -fastq_maxee 1.0). Filtered reads were then de-duplicated into unique reads (command: fastx_uniques), which were clustered into OTUs (command: cluster_otu, option: -minsize 2) with a 97% identity. Against the OTU representative sequences, all reads were aligned using the usearch_global command with an identity cutoff 0.97. Based on the resulting OTU table, frequencies of all OTUs in each sample were calculated. To analyze beta diversity, a distance matrix of the OTU representative sequences was calculated (command: calc_distmx, option: -maxdist 1.0) for constructing a phylogenetic tree using the command cluster_aggd with average linkage. With the tree and OTU table, beta diversity was calculated using the python script “beta_diversity_through_plots.py” in QIIME [30] (v1.9), which applied the UniFrac [20] metrics for measuring distance between samples.

## Supplementary information

**Additional file 1.** The file contains five supplementary figures and four supplementary tables.

## Data Availability

The Illumina PE data supporting the conclusions of this article are available in the NCBI Sequence Read Archive (BioProject ID: PRJNA544825).

## Abbreviations

PE: Paired-end
NGS: Next-generation sequencing
rRNA/rDNA: Ribosomal RNA/DNA
R1/2: First/second read
RevComp: Reverse complement
SRA: Sequence read archive
BTOP/TOP: Local/global alignment based top hit method for classification
OTU: Operational taxonomic unit
OC: Over-classification
UC: Under-classification
MC: Misclassification
TP: True positive
PCR: Polymerase chain reaction
DJ: Direct-joining
IO: Inside-out

## References

[1] Handelsman J (2004). Metagenomics: application of genomics to uncultured microorganisms. Microbiol Mol Biol Rev.

[2] Streit WR, Schmitz RA (2004). Metagenomics--the key to the uncultured microbes. Curr Opin Microbiol.

[3] Metzker ML (2010). Sequencing technologies - the next generation. Nat Rev Genet.

[4] Cole JR, Wang Q, Fish JA, Chai B, McGarrell DM, Sun Y, Brown CT, Porras-Alfaro A, Kuske CR, Tiedje JM (2014). Ribosomal database project: data and tools for high throughput rRNA analysis. Nucleic Acids Res.

[5] McDonald D, Price MN, Goodrich J, Nawrocki EP, DeSantis TZ, Probst A, Andersen GL, Knight R, Hugenholtz P (2012). An improved Greengenes taxonomy with explicit ranks for ecological and evolutionary analyses of bacteria and archaea. ISME J.

[6] Johnson JS, Spakowicz DJ, Hong BY, Petersen LM, Demkowicz P, Chen L, Leopold SR, Hanson BM, Agresta HO, Gerstein M, et al. Evaluation of 16S rRNA gene sequencing for species and strain-level microbiome analysis. Nat Commun. 2019;10(1):5029. 10.1038/s41467-019-13036-1 PMC6834636 31695033

[7] Gardner Allison M., Muturi Ephantus J., Allan Brian F. (2018). Discovery and exploitation of a natural ecological trap for a mosquito disease vector. Proceedings of the Royal Society B: Biological Sciences.

[8] Chen J, Toyomasu Y, Hayashi Y, Linden DR, Szurszewski JH, Nelson H, Farrugia G, Kashyap PC, Chia N, Ordog T (2016). Altered gut microbiota in female mice with persistent low body weights following removal of post-weaning chronic dietary restriction. Genome Med.

[9] Leff JW, Jones SE, Prober SM, Barberan A, Borer ET, Firn JL, Harpole WS, Hobbie SE, Hofmockel KS, Knops JM (2015). Consistent responses of soil microbial communities to elevated nutrient inputs in grasslands across the globe. Proc Natl Acad Sci U S A.

[10] Soergel DA, Dey N, Knight R, Brenner SE (2012). Selection of primers for optimal taxonomic classification of environmental 16S rRNA gene sequences. ISME J.

[11] Werner JJ, Zhou D, Caporaso JG, Knight R, Angenent LT (2012). Comparison of Illumina paired-end and single-direction sequencing for microbial 16S rRNA gene amplicon surveys. ISME J.

[12] Edgar RC (2018). Accuracy of taxonomy prediction for 16S rRNA and fungal ITS sequences. PeerJ.

[13] Wood DE, Lu J, Langmead B (2019). Improved metagenomic analysis with kraken 2. Genome Biol.

[14] Jeraldo P, Kalari K, Chen X, Bhavsar J, Mangalam A, White B, Nelson H, Kocher JP, Chia N (2014). IM-TORNADO: a tool for comparison of 16S reads from paired-end libraries. PLoS One.

[15] Parikh HI, Koparde VN, Bradley SP, Buck GA, Sheth NU (2016). MeFiT: merging and filtering tool for illumina paired-end reads for 16S rRNA amplicon sequencing. BMC Bioinformatics.

[16] Edgar RC (2010). Search and clustering orders of magnitude faster than BLAST. Bioinformatics.

[17] Wang Q, Garrity GM, Tiedje JM, Cole JR (2007). Naive Bayesian classifier for rapid assignment of rRNA sequences into the new bacterial taxonomy. Appl Environ Microbiol.

[18] Edgar RC. SINTAX: a simple non-Bayesian taxonomy classifier for 16S and ITS sequences. bioRxiv. 2016. 10.1101/074161.

[19] Klindworth A, Pruesse E, Schweer T, Peplies J, Quast C, Horn M, Glockner FO (2013). Evaluation of general 16S ribosomal RNA gene PCR primers for classical and next-generation sequencing-based diversity studies. Nucleic Acids Res.

[20] Lozupone C, Knight R (2005). UniFrac: a new phylogenetic method for comparing microbial communities. Appl Environ Microbiol.

[21] Edgar RC (2013). UPARSE: highly accurate OTU sequences from microbial amplicon reads. Nat Methods.

[22] Callahan BJ, McMurdie PJ, Rosen MJ, Han AW, Johnson AJ, Holmes SP (2016). DADA2: high-resolution sample inference from Illumina amplicon data. Nat Methods.

[23] Werner JJ, Koren O, Hugenholtz P, DeSantis TZ, Walters WA, Caporaso JG, Angenent LT, Knight R, Ley RE (2012). Impact of training sets on classification of high-throughput bacterial 16s rRNA gene surveys. ISME J.

[24] Teo SM, Mok D, Pham K, Kusel M, Serralha M, Troy N, Holt BJ, Hales BJ, Walker ML, Hollams E (2015). The infant nasopharyngeal microbiome impacts severity of lower respiratory infection and risk of asthma development. Cell Host Microbe.

[25] Alnahas S, Hagner S, Raifer H, Kilic A, Gasteiger G, Mutters R, Hellhund A, Prinz I, Pinkenburg O, Visekruna A (2017). IL-17 and TNF-alpha are key mediators of Moraxella catarrhalis triggered exacerbation of allergic airway inflammation. Front Immunol.

[26] O'Connor GT, Lynch SV, Bloomberg GR, Kattan M, Wood RA, Gergen PJ, Jaffee KF, Calatroni A, Bacharier LB, Beigelman A (2018). Early-life home environment and risk of asthma among inner-city children. J Allergy Clin Immunol.

[27] Durack J, Lynch SV, Nariya S, Bhakta NR, Beigelman A, Castro M, Dyer AM, Israel E, Kraft M, Martin RJ (2017). Features of the bronchial bacterial microbiome associated with atopy, asthma, and responsiveness to inhaled corticosteroid treatment. J Allergy Clin Immunol.

[28] Huang YJ, Nelson CE, Brodie EL, Desantis TZ, Baek MS, Liu J, Woyke T, Allgaier M, Bristow J, Wiener-Kronish JP (2011). Airway microbiota and bronchial hyperresponsiveness in patients with suboptimally controlled asthma. J Allergy Clin Immunol.

[29] Huang W, Li L, Myers JR, Marth GT (2012). ART: a next-generation sequencing read simulator. Bioinformatics.

[30] Caporaso JG, Kuczynski J, Stombaugh J, Bittinger K, Bushman FD, Costello EK, Fierer N, Pena AG, Goodrich JK, Gordon JI (2010). QIIME allows analysis of high-throughput community sequencing data. Nat Methods.

