# Supplementary material for: Joining Illumina paired-end reads for classifying phylogenetic marker sequences
Source: BMC Bioinformatics. 2020 Mar 14;21:105. doi: 10.1186/s12859-020-3445-6 (PMC7071698; doi:10.1186/s12859-020-3445-6)
Supplement: Supplementary file 1 — Additional file 1. The file contains five supplementary figures and four supplementary tables. [file 12859_2020_3445_MOESM1_ESM.docx]

**Additional file 1**

**Supplementary Figures**


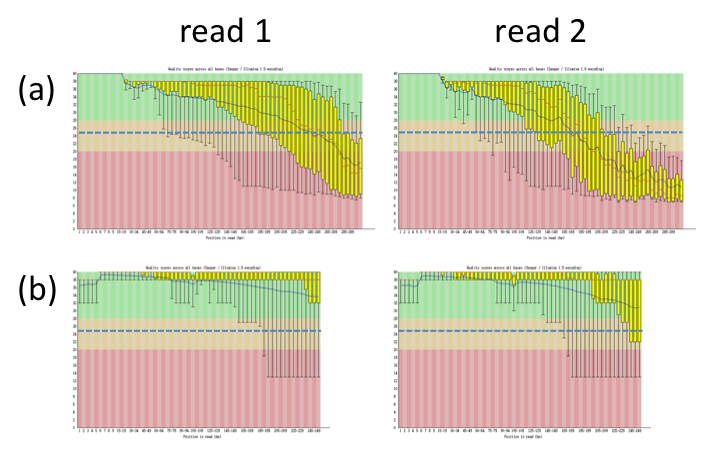


Figure S1. Quality profiles of (a) our real MiSeq 2×300 bp and (b) NCBI SRA HiSeq 2×250 bp reads obtained using FastQC. The blue solid lines indicate mean quality and the blue dashed lines stand for Q25.

| 1. True positive rate   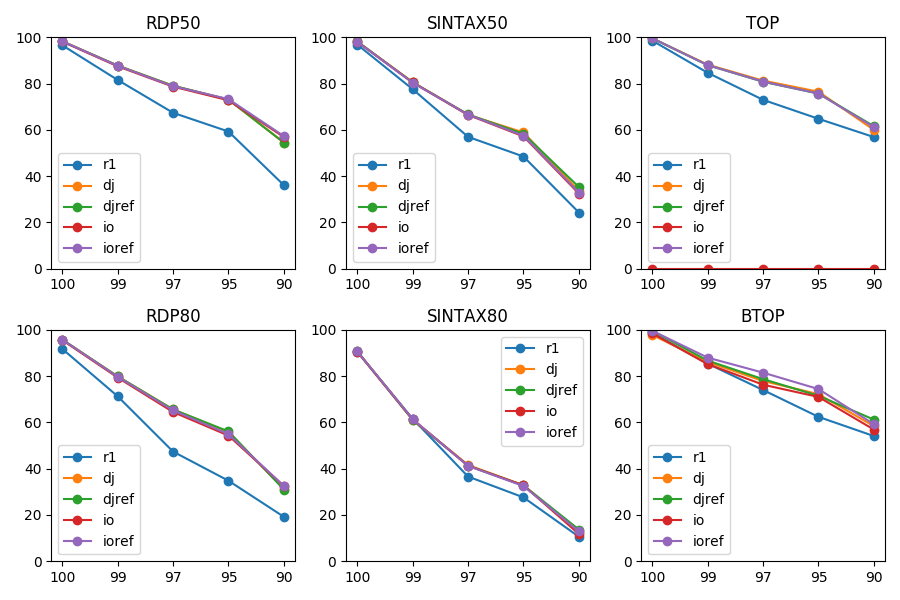 | 1. Under-classification rate   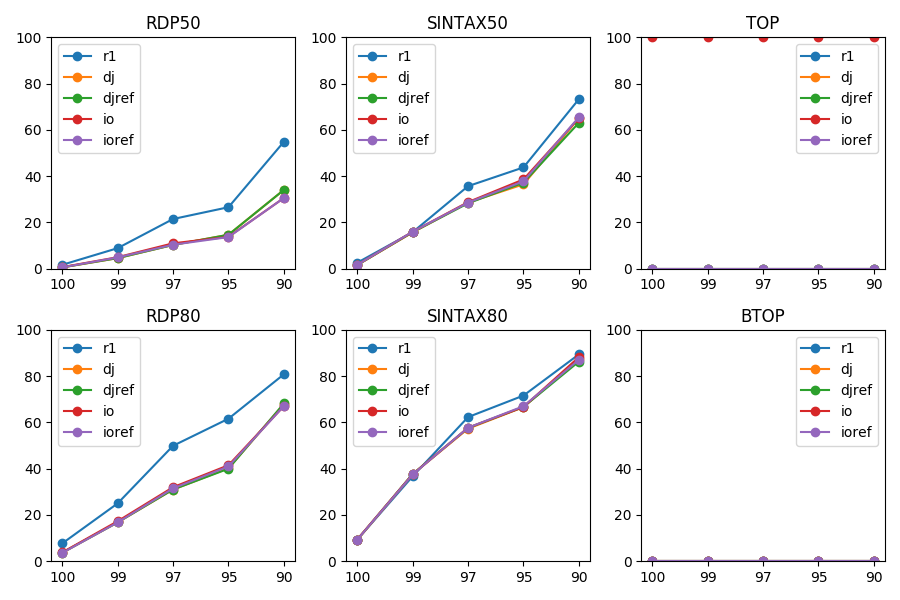 |
| --- | --- |
| 1. Over-classification rate   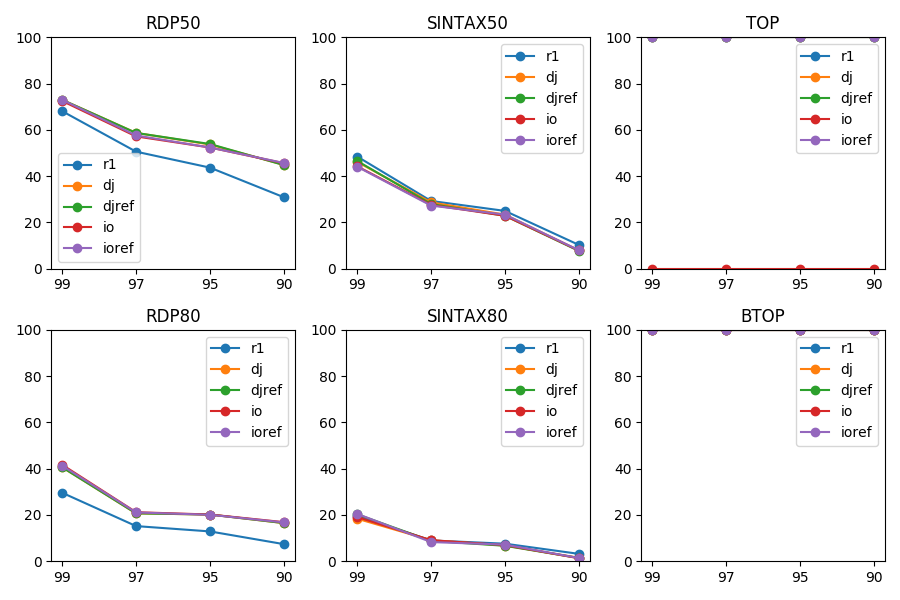 | 1. Misclassification rate   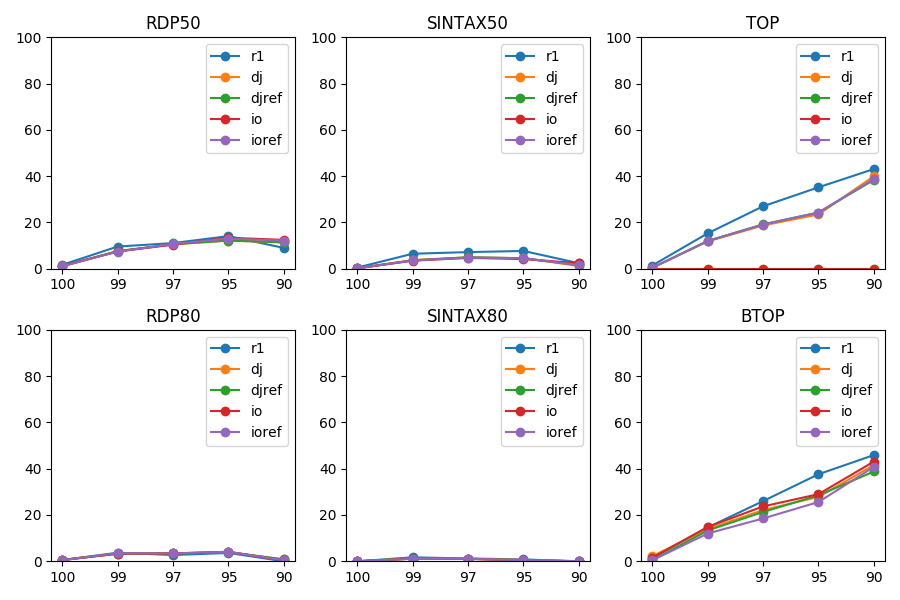 |

Figure S2. Performance metrics (y-axis) for genus prediction of different types of simulated MiSeq data by six classification methods at different top-hit identities (x-axis) for the V3-V5 primer pair.

| 1. True positive rate   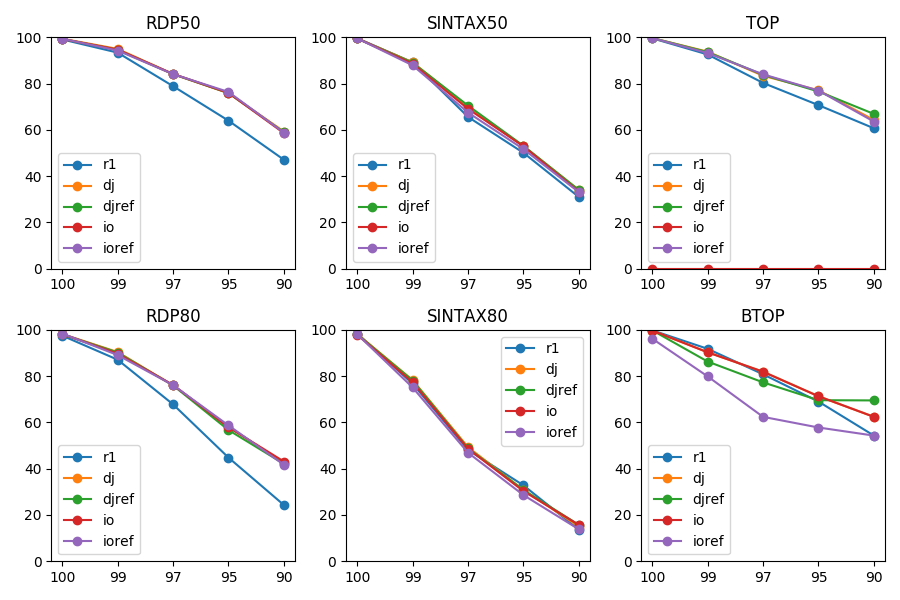 | 1. Under-classification rate   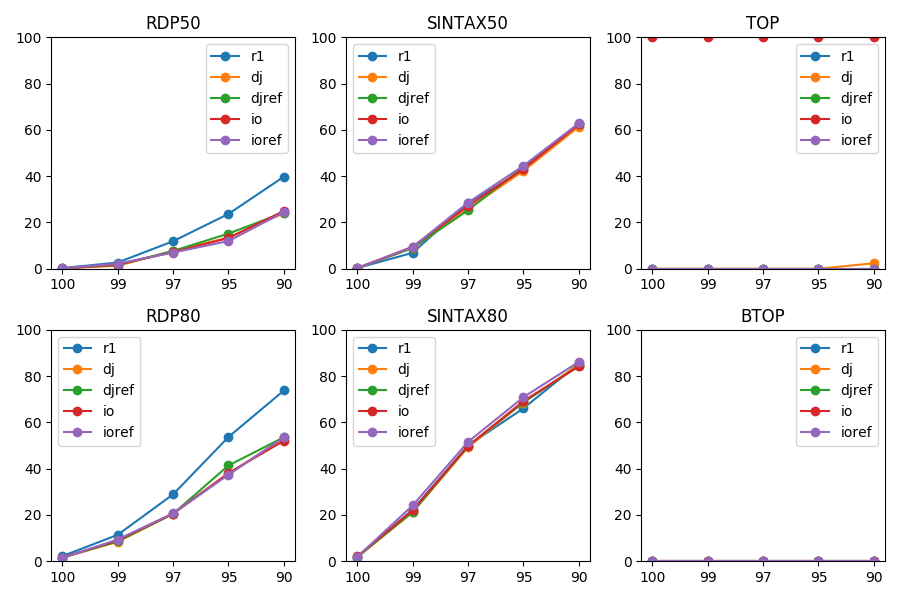 |
| --- | --- |
| 1. Over-classification rate   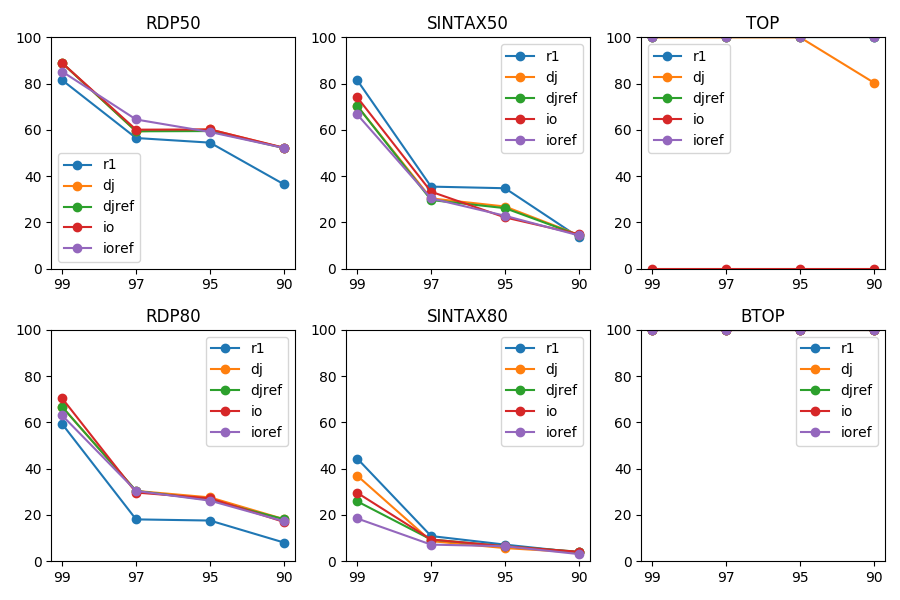 | 1. Misclassification rate   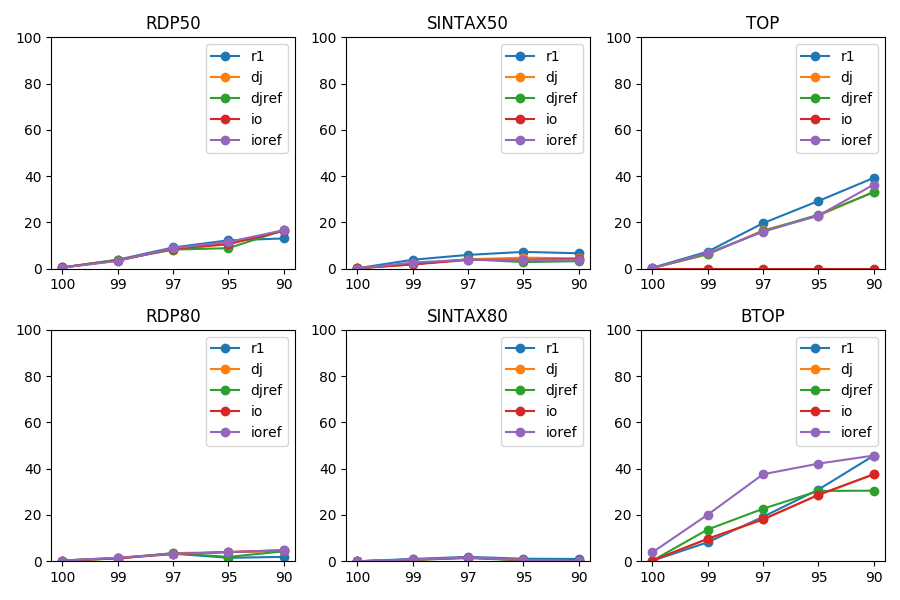 |

Figure S3. Performance metrics (y-axis) for genus prediction of different types of simulated MiSeq data by six classification methods at different top-hit identities (x-axis) for the V1-V3 primer pair.


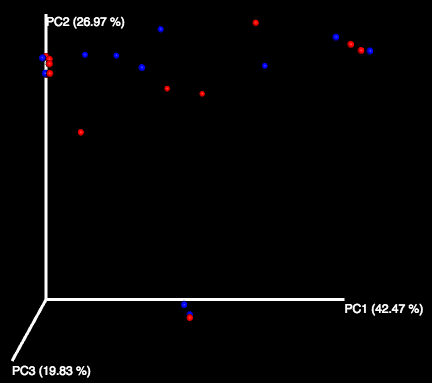


Figure S4. Principal coordinate analysis using weighted UniFrac distances between the samples during asthma attack (red) and in the recovery phase (blue).


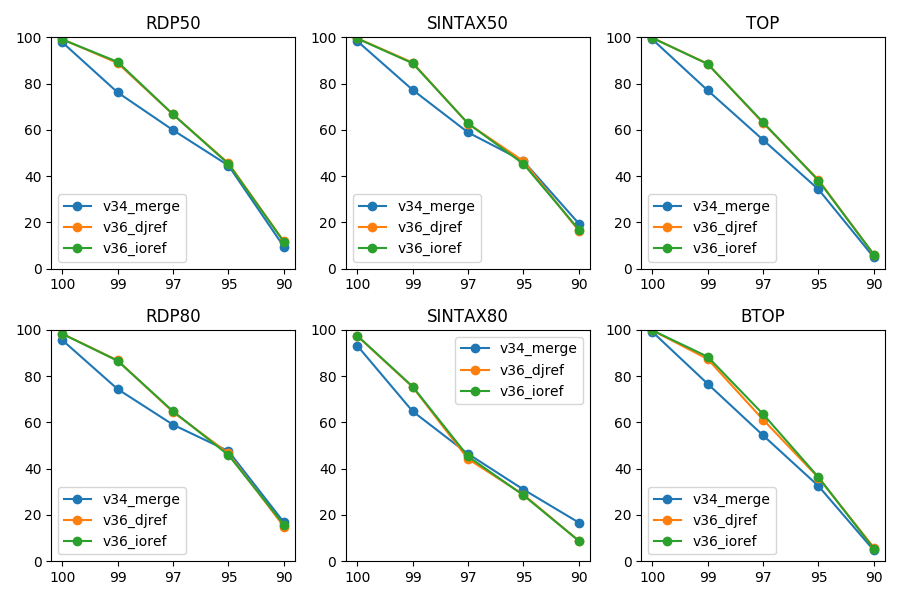


Figure S5. Accuracies (y-axis) of classifying merged reads of the V3-V4 amplicons, and directly joined and inside-out reads of the V3-V6 amplicons to the genus level at different top-hit identities (x-axis) by six classification methods using error-free MiSeq 2×300 bp data.

**Supplementary Tables**

| patient | V1-V3 attack | V1-V3 recovery | V3-V5 attack | V3-V5 recovery |
| --- | --- | --- | --- | --- |
| P1 | 232834; 106521 | 92717; 40760 | 18742; 67 | 2796; 150 |
| P2 | 177653; 85162 | 176750; 84420 | 4579; 899 | 11949; 5768 |
| P3 | 253322; 116256 | 228222; 103942 | 4663; 211 | 7938; 1399 |
| P4 | 175690; 86412 | 237446; 118171 | 26947; 8454 | 11894; 2925 |
| P5 | 230340; 111047 | 55306; 23257 | 16984; 5854 | 8785; 72 |
| P6 | 231706; 108459 | 106585; 53762 | 20029; 3684 | 56137; 5120 |
| P7 | 165959; 95249 | 69690; 32792 | 12601; 115 | 7976; 3609 |
| P8 | 31997; 13242 | 67902; 30994 | 8062; 1447 | 2574; 486 |
| P9 | 127839; 63587 | 104011; 59300 | 9693; 2227 | 4118; 179 |
| P10 | 134483; 63576 | 123416; 59112 | 11312; 3058 | 4093; 1293 |
| P11 | 194262; 104083 | 122585; 58261 | 20797; 922 | 2678; 364 |
| P12 | 176354; 97358 | 42137; 23674 | 41071; 462 | 3751; 160 |

Table S1. Numbers of PEs and merged reads of the V1-V3 and V3-V5 data of the twelve asthmatic children during asthma attack and in the recovery phase.

| Project ID | No. of experiments in the project | Representative experiment | % of PE reads that could be merged |
| --- | --- | --- | --- |
| SRP200733 | 10 | SRX5985117 | 0.28 |
| SRP100723 | 264 | SRX2589613 | 1.65 |
| SRP045730 | 7 | SRX685535 | 3.79 |
| SRP226558 | 111 | SRX7032607 | 7.35 |
| SRP136405 | 3 | SRX3843879 | 13.68 |
| SRP159477 | 300 | SRX4635302 | 14.80 |
| SRP188599 | 156 | SRX5528992 | 15.80 |
| SRP074626 | 84 | SRX2241955 | 18.52 |
| SRP082155 | 273 | SRX2023216 | 30.63 |
| SRP064512 | 118 | SRX1307092 | 32.92 |
| SRP110772 | 224 | SRX2977977 | 33.77 |
| SRP069981 | 2 | SRX1583067 | 42.45 |
| SRP108278 | 1 | SRX2869140 | 59.64 |
| SRP153935 | 1 | SRX4395263 | 81.79 |
| SRP152214 | 23 | SRX4342766 | 82.56 |
| SRP117913 | 1 | SRX3195510 | 85.99 |
| SRP166816 | 1 | SRX4935398 | 86.85 |
| SRP065068 | 226 | SRX1360988 | 86.96 |
| SRP166705 | 1 | SRX4927952 | 87.78 |
| SRP162142 | 1 | SRX4717167 | 89.25 |
| SRP057511 | 37 | SRX1004572 | 89.57 |
| SRP149964 | 1 | SRX4182583 | 90.70 |
| SRP152889 | 768 | SRX4371116 | 91.26 |
| SRP114951 | 25 | SRX3067447 | 95.15 |
| SRP125133 | 1 | SRX3400284 | 95.89 |
| SRP111625 | 2 | SRX2996885 | 97.44 |
| ERP009544 | 12 | ERX941623 | 97.61 |
| SRP182765 | 1 | SRX5305177 | 97.66 |
| SRP167862 | 1 | SRX4978622 | 97.91 |
| SRP218505 | 1 | SRX6723040 | 98.67 |
| SRP219247 | 1 | SRX6757474 | 98.82 |
| SRP081324 | 1 | SRX2013860 | 99.17 |
| SRP131935 | 14 | SRX3637932 | 99.69 |

Table S2. Percentage of PE reads that could be merged for the 33 metagenomics projects using the V3-V5 primer pair in NCBI Sequence Read Archive. For each project, one representative data (the last piece of query) was downloaded to estimate the percentage of merged data using USEARCH with a 25% maximal mismatch rate and minimal overlap as 16 bp.

| OTU | P1 | P2 | P3 | P4 | P5 | P6 | P7 | P8 | P9 | P10 | P11 | P12 | genus |
| --- | --- | --- | --- | --- | --- | --- | --- | --- | --- | --- | --- | --- | --- |
| Otu1 | -3.65 | 0.13 | -9.23 | -2.40 | 61.79 | -0.12 | -37.73 | 0.45 | 69.74 | -1.81 | -0.05 | -3.82 | Rhodococcus |
| Otu7 | -0.45 | 1.01 | -1.14 | 4.17 | 7.49 | -0.01 | -2.73 | 0.02 | 6.63 | 0.53 | 0.02 | 0.03 | Sphingomonas |
| Otu119 | -0.23 | -1.28 | -0.55 | 1.92 | 4.01 | -0.01 | -2.78 | 0.05 | 3.38 | 2.21 | -0.01 | -0.46 | Rhodococcus |
| Otu11 | 0.01 | 1.02 | -0.04 | 1.02 | 2.24 | 0.00 | -0.29 | 0.00 | 0.80 | -0.32 | 0.00 | -0.06 | Bradyrhizobium |
| Otu13 | -1.51 | -0.24 | -0.08 | -2.55 | 0.04 | 25.17 | -4.19 | 0.35 | -0.06 | -0.19 | 0.00 | 0.02 | Staphylococcus |
| Otu12 | -0.09 | -0.49 | -0.05 | -1.41 | 1.65 | 0.00 | -0.43 | 0.01 | 1.12 | -0.08 | 0.00 | -0.02 | Delftia |
| Otu4 | -0.03 | 0.93 | -33.19 | -0.01 | -0.05 | 54.37 | -0.09 | -30.79 | -0.06 | -0.03 | -21.42 | 0.00 | Corynebacterium |
| Otu5 | 0.00 | 0.18 | 0.02 | -0.01 | -0.01 | -0.23 | 0.00 | 0.40 | -97.25 | -0.01 | 4.03 | -88.92 | Moraxella |
| Otu15 | 0.00 | -0.01 | -0.01 | -0.08 | 0.42 | -0.08 | -0.05 | 0.21 | 3.47 | 0.91 | -0.11 | -0.02 | Streptococcus |
| Otu35 | 0.00 | 0.00 | 0.00 | -0.03 | -0.06 | -0.01 | 0.09 | 0.03 | 1.32 | -0.04 | -0.03 | -0.01 | Streptococcus |
| Otu6 | -0.02 | 0.09 | 40.52 | -0.01 | -0.07 | -16.42 | -0.07 | 46.79 | -0.22 | -0.01 | -4.84 | 0.03 | Dolosigranulum |
| Otu28 | 0.01 | 0.90 | -0.01 | -0.01 | -0.03 | -0.03 | 0.04 | 0.04 | 1.23 | 0.01 | -0.03 | 0.02 | Rothia |
| Otu42 | 0.00 | 0.00 | 0.00 | 0.00 | 0.00 | 0.00 | 0.04 | 0.00 | 0.00 | 2.09 | 0.00 | 0.00 | Lautropia |
| Otu14 | 0.00 | 0.00 | 0.00 | 5.15 | 0.00 | 0.00 | 0.00 | 0.00 | 0.00 | 0.00 | 0.00 | 0.00 | Streptococcus |
| Otu24 | 0.00 | -0.01 | -0.01 | 0.00 | 0.00 | 0.00 | 1.72 | 0.00 | 0.00 | 0.00 | 0.00 | 0.00 | Neisseria |
| Otu3 | 6.30 | 0.95 | 0.09 | 1.67 | -95.38 | -0.15 | -0.15 | -0.03 | 0.30 | 0.03 | -0.05 | -0.04 | Staphylococcus |
| Otu39 | -0.02 | 0.17 | -0.01 | 0.21 | 1.73 | 0.41 | 0.00 | -6.62 | 3.42 | 0.26 | -0.01 | 0.01 | Corynebacterium |
| Otu22 | 0.00 | 2.09 | 0.00 | 0.00 | 0.00 | 0.00 | -0.38 | 0.00 | 0.00 | 0.00 | 0.00 | 0.00 | Simonsiella |
| Otu16 | 0.00 | 0.01 | 0.00 | 0.01 | 0.01 | 0.10 | 0.00 | -11.90 | 1.18 | 0.00 | 0.00 | 0.00 | Corynebacterium |
| Otu2 | -0.01 | 0.01 | 4.34 | 0.00 | -0.08 | -0.39 | 70.56 | 0.72 | 0.55 | 0.00 | 28.72 | 92.71 | Moraxella |
| Otu26 | 0.00 | 0.00 | 0.00 | -1.53 | 1.03 | 0.00 | 0.00 | 0.00 | 0.00 | 0.00 | 0.00 | 0.00 | Streptophyta |
| Otu18 | 0.02 | 0.00 | 0.00 | 3.27 | 0.00 | 0.00 | 0.00 | 0.00 | 0.00 | 0.00 | 0.00 | 0.00 | GpI |
| Otu32 | 0.00 | 0.00 | 0.00 | 0.00 | 1.44 | 0.00 | 0.00 | 0.00 | 0.00 | 0.00 | 0.00 | 0.00 | Gordonia |
| Otu20 | 0.00 | 0.00 | 0.00 | 0.00 | 2.63 | 0.00 | 0.00 | 0.00 | 0.00 | 0.00 | 0.00 | 0.00 | Corynebacterium |

Table S3. Frequency difference (in %) of OTUs derived from the directly joined reads between attack and recovery states for nose samples of the twelve asthmatic children. Only OTUs with ≥1% difference in at least one sample are shown.

| Read | RDP50 | RDP80 | SINTAX50 | SINTAX80 | TOP | BTOP |
| --- | --- | --- | --- | --- | --- | --- |
| v34_merge | 57.62 | 58.68 | 60.12 | 50.42 | 54.24 | 53.5 |
| v36_djref | 62.44 | 62.24 | 62.86 | 50.9 | 59.18 | 58.08 |
| v36_ioref | 62.42 | 62.28 | 62.66 | 51.12 | 59.16 | 58.7 |

Table S4. Mean accuracies of classifying the perfect V3-V4 amplicons, and error-free directly joined and inside-out reads of the V3-V6 amplicons to the genus level by six classification methods.
